# Medical Items

**Published:** 1892-09

**Authors:** 


### *MEDICAL ITEMS.*

### NOTICE.

Owing to the loss of many JOURNALS through their wrappers being torn off by rough handling in the postoffice—when "mailed flat," as heretofore—we are compelled to have them "rolled." We hope to make arrangements to safely mail them "flat" again.

---

THE JOURNAL last month presented the Grady Hospital with a handsome faradic battery.

---

THE inventor of the hypodermic syringe, Dr. Pravaz, died last month at Lyons, France. His little instrument is still known in France as the *Seringue de Pravaz.*

---

THE sixteenth annual meeting of the American Dermatological Association will be held at the Pequot House, New London, Conn., September 13th, 14th and 15th. Dr. E. B. Bronson, President; Dr. Geo. T. Jackson, Secretary.

---

THE August number of the *Medical Bulletin* announces that the "ATLANTA MEDICAL AND SURGICAL JOURNAL has passed into the hands of Drs. Hutchins and Grandy."

Our dear Philadelphia contemporary is quite correct, but oh, so terribly behind time! Still we cannot expect too much haste in Philadelphia.

---

THE latest novelty in the suicide business has been introduced by Marguerite Borel of Paris. Her lover deserted her; whereupon Marguerite, with her feelings terribly lacerated, purchased in different drugstores a large supply of leeches. Then she went to her apartments, undressed, and applied the leeches all over her body. The leeches cheerfully rendered the necessary assistance and Marguerite died in a few hours.

DR. CHARLES E. SAJOUS, of Philadelphia, editor of *The Medical Annual*, has been appointed a Knight of the Legion of Honor of France by President Carnot, for his services to the French colony and to science, as an American of French descent. Dr. Sajous went to Paris last May to translate his *Medical Annual*, and he expects to make Paris his home for the next three years.

---

IN the absence of the regular monthly letter from our New York correspondent, Dr. J. Arthur Childs furnishes us with an interesting letter on the treatment of genito-urinary diseases in the metropolis. Dr. Childs has returned from New York, where he pursued a thorough course in genito-urinary diseases. He resumes his practice in Atlanta, which will hereafter be confined to the above named branch.

---

KEELEY is now in England trying industriously to get his methods established there. His efforts, however, have been disappointing. He had almost succeeded at one time in getting an indorsement from the Church of England Temperance Society. A large meeting was held, and Keeley was getting in his good work, when at the last moment his scheme was aborted by some regular physicians who had the meeting postponed *sine die*.

---

WE have received a copy of an abstract of the proceedings of the last meeting of the Tri-State Medical Society of Alabama, Georgia and Tennessee, which has just been issued from the office of the secretary. It contains an abstract of all the papers read and of the discussions thereon. The advantage of an abstract of this kind is that it is not necessary to read through all the papers to get the leading ideas advanced by the various authors. Many of the papers have been published in the journals and reprints that can be obtained of most of them by addressing the authors.

The abstract will be sent free by addressing Dr. Frank Trester Smith, Chattanooga, Tenn.

The next meeting of the Society will be held in Chattanooga, beginning Tuesday, October 25th.

OUT in Omaha, Kansas, resided a quack of the first magnitude, one C. Gee Wo, who has lately been tried and convicted for violating a State act relative to the legitimate practice of medicine. In fixing the fine Judge Davis said: "I wish that I could make the fine $5,000, for I believe that you are a plague to the community as a quack doctor. Take the witnesses who were called in your case; they are a sample of the class of people who patronize you, half-witted and simple-minded, with scarcely memory enough to tell what they were troubled with. They are like the people who followed false prophets, believing that there was some miraculous power in their nostrums. We have heard of some of them dying in the hospital for the lack of a little simple surgery by reason of the faith that they had in this juggler in medicine."

The court imposed a fine of $300 and costs—the limit—and required C. Gee Wo to give a bond of $2,000 to be of good behavior for two years and refrain from practicing medicine.

---

THE New York medical journals have had practically nothing to say with reference to the late troubles between Tammany Hall and the Board of Health of the city. This board, of course, is controlled, as everything else is, by Tammany Hall, which reserves and exercises the right of appointment and removal according to its own sweet pleasure. We learn that efficient and experienced officers have been removed without cause or pretext. After a recent instance of this sort Prof. Janeway resigned from the Advisory Board and resigned also his professorship in Bellevue Hospital Medical College and as visiting physician to Bellevue Hospital. In this he was promptly followed by Drs. Prudden, O'Dwyer, Derby, Jacobi, Stimpson and others. Our distinguished and learned friend, Dr. Geo. F. Shrady, of the *Medical Record*, alone remained on the board. Parenthetically we may observe that Dr. Shrady's son is inspector in the vaccination bureau. Sometimes silence is golden.

